# KSHV-encoded LANA bypasses transcriptional block through the stabilization of RNA Pol II in hypoxia

**DOI:** 10.1128/mbio.02774-23

**Published:** 2023-12-14

**Authors:** Dipayan Bose, Rajnish Kumar Singh, Erle S. Robertson

**Affiliations:** 1 Tumor Virology Program, Department of Otorhinolaryngology-Head and Neck Surgery, Perelman School of Medicine, University of Pennsylvania, Philadelphia, Pennsylvania, USA; Princeton University, Princeton, New Jersey, USA

**Keywords:** hypoxia, KSHV, lytic reactivation, RNA Polymerase II, ubiquitination

## Abstract

Hypoxia results from an insufficient supply of oxygen, which results in physiological stress in biological systems. Cells respond to this unfavorable condition by reducing critical cellular functions, which include replication, transcription, and translation. Oncogenic Kaposi sarcoma-associated virus (KSHV) undergoes lytic reactivation in hypoxic conditions. Furthermore, reactivation requires the synthesis of specific viral proteins, both structural and non-structural. Therefore, the virus manipulates the cellular microenvironment to ensure efficient functioning of the cellular transcription machinery, including RNA polymerase II (Pol II), by protecting it from hypoxia-mediated degradation. In this study, we demonstrated that hypoxia induces degradation of RNA Pol II via ubiquitination mediated by the NEDD4 E3 ligase, which results in transcription inhibition. However, in KSHV-infected cells during hypoxia, the virus-encoded latency-associated nuclear antigen interacts with NEDD4, inhibiting its E3 ubiquitin ligase activity and requires complex formation with hypoxia inducible factor HIF1α. This activity inhibits the polyubiquitination of RNA Pol II to maintain steady transcription levels necessary for the synthesis of the essential viral genes needed for successful lytic reactivation.

**IMPORTANCE:**

Hypoxia can induce the reactivation of Kaposi sarcoma-associated virus (KSHV), which necessitates the synthesis of critical structural proteins. Despite the unfavorable energetic conditions of hypoxia, KSHV utilizes mechanisms to prevent the degradation of essential cellular machinery required for successful reactivation. Our study provides new insights on strategies employed by KSHV-infected cells to maintain steady-state transcription by overcoming hypoxia-mediated metabolic stress to enable successful reactivation. Our discovery that the interaction of latency-associated nuclear antigen with HIF1α and NEDD4 inhibits its polyubiquitination activity, which blocks the degradation of RNA Pol II during hypoxia, is a significant contribution to our understanding of KSHV biology. This newfound knowledge provides new leads in the development of novel therapies for KSHV-associated diseases.

## INTRODUCTION

Transcription is the initial step in gene expression, using genetic information from an open reading frame (ORF) or gene to generate mRNA that is translated into a functional protein (1). RNA polymerase (RNAP), a key enzyme, reads a single-stranded DNA template, synthesizing the complementary RNA strand in the 5′ to 3′ direction, locally unwinding the DNA (1, 2). This process requires transcription factors and a mediator complex, which binds to the DNA’s promoter region before RNA polymerase can initiate DNA unwinding (3). RNA polymerase not only initiates transcription but also guides nucleotides, aids attachment and elongation, and possesses proofreading and termination recognition capabilities (4).

RNA polymerase, abundant in all organisms and some viruses, exists as a multi-subunit RNAP or a single-subunit ssSNAP, depending on the organism (5). Multi-subunit RNAP is found in bacteria, archaea, and eukaryotes, sharing a common core structure and mechanism (6). Single-subunit ssSNAP is found in phages, eukaryotic chloroplasts, and mitochondria, with similarities to modern DNA polymerases (7). However, eukaryotic and archaeal RNAPs have more subunits and distinct regulation compared to bacterial enzymes (8).

Eukaryotes have multiple types of nuclear RNAP, each responsible for the synthesis of a distinct subset of RNA. RNA polymerase I synthesizes a pre-rRNA 45S (35S in yeast), which matures and forms the major RNA sections of the ribosome (9). RNA polymerase II synthesizes precursors of mRNAs and most snRNA and microRNAs (10). RNA polymerase III synthesizes tRNAs, rRNA 5S, and other small RNAs found in the nucleus and cytosol (11). RNA polymerase IV and V found in plants are less understood; they synthesize siRNA (12). In addition to the ssSNAPs, chloroplasts also encode and use a bacteria-like RNAP (13).

RNA polymerase II (RNAP II and RNA Pol II) is a 550 kDa complex consisting of 12 subunits (14). The subunits include DNA-directed RNA polymerase II subunit RPB (1–12, 15). Among these 12 subunits, DNA-directed RNA polymerase II subunit RPB1 is encoded by the POLR2A gene and is the largest subunit of RNA polymerase II. It contains a carboxy-terminal domain composed of up to 52 heptapeptide repeats (YSPTSPS) that are essential for polymerase activity (10). In combination with several other polymerase subunits, the RPB1 subunit forms the DNA-binding domain of the polymerase and binds to a groove from which the DNA template is transcribed into RNA (16).

Hypoxia, a condition marked by low oxygen levels, can have significant impacts on both physiological and pathological processes, including development, ischemia, stroke, and cancer (17, 18). It induces changes in gene expression by affecting both transcription and translation (19). When exposed to hypoxic conditions, cells reduce their overall rate of messenger RNA (mRNA) generation and translation (20–22). However, the effects on individual mRNA species can vary greatly, with some even being stimulated under these conditions (23). This regulation of translation in response to hypoxia can lead to differential gene expression. The ability of cells to control transcription and translation during hypoxia is crucial for their survival.

Several viruses, including KSHV, are known to undergo lytic reactivation under stress conditions such as hypoxia (24). During reactivation, several viral encoded structural genes, including capsid and envelope proteins, are essential for the generation of progeny particles (25). Therefore, the aim of the present study was to determine the mechanism of sustained RNA transcription in the hypoxic microenvironment specifically in the context of KSHV infection.

Notably, transcription by RNA Pol II is not a smooth, continuous process. Many factors affect the processivity of RNA Pol II. *cis*-acting factors like topological strains and chromatin 3D structures can influence the transcription process (26). Similarly, *trans*-acting factors like the availability of nucleotides, depletion of dNTPs, and the overall shortage of available energy lead to stalling of the RNA Pol II on the DNA template (27). The stalled RNA Pol II is then polyubiquitinylated leading to degradation through the proteasomal degradation pathway (28, 29).

The occurrence of hypoxia can stimulate the reactivation of KSHV, a process that requires the synthesis of essential structural proteins. Although hypoxia is energetically unfavorable (17), KSHV has evolved mechanisms that prevent the degradation of necessary components of cellular machinery essential for successful reactivation. Our investigation focuses on the strategy utilized by KSHV to protect cellular RNA Pol II from degradation induced by hypoxia, thereby promoting successful lytic reactivation.

## RESULTS

### KSHV can influence the rate of transcription during hypoxia

**Fig 1:**
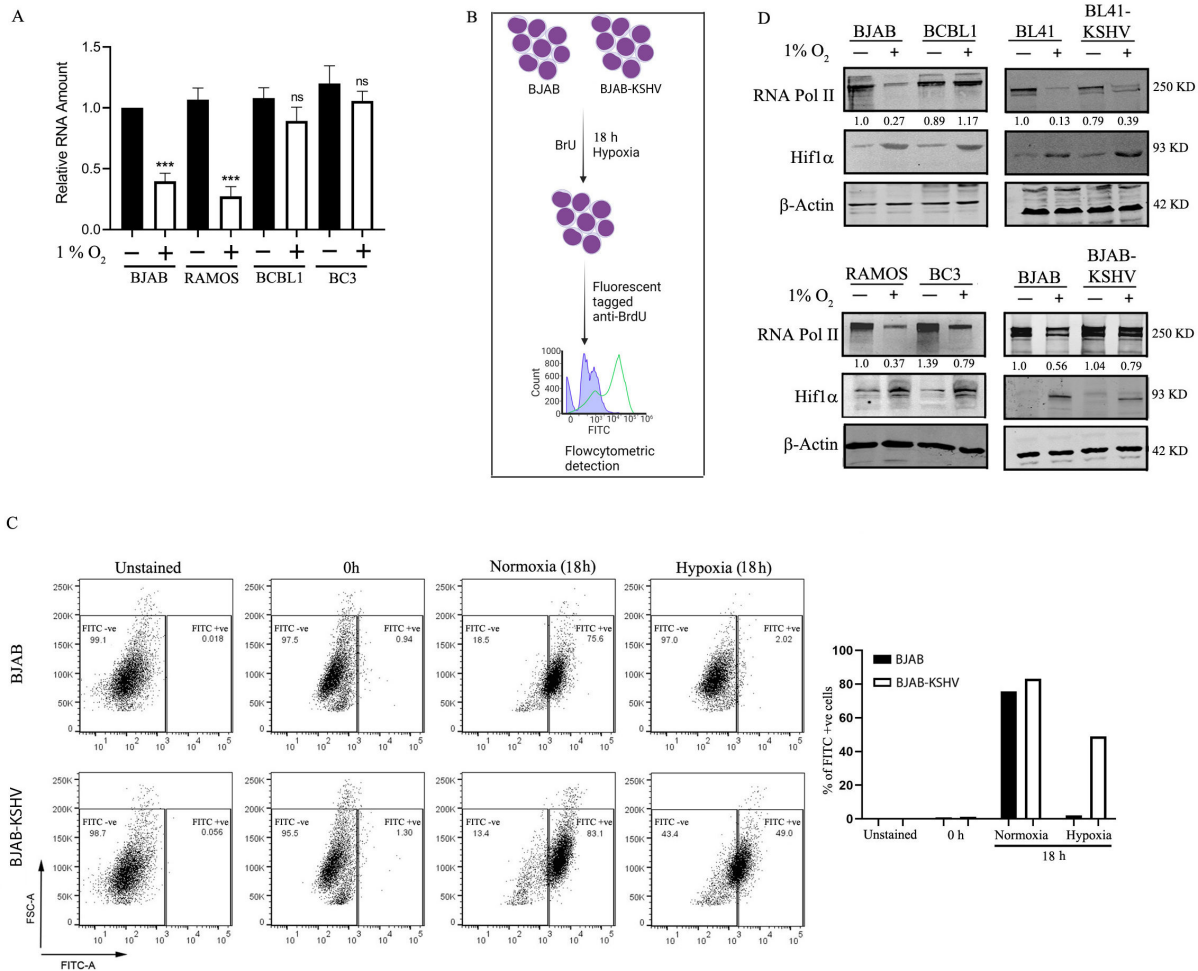
Effect of hypoxia on transcription in KSHV-positive and negative cells. (**A**) Total mRNA was isolated from 10 million KSHV-negative BJAB, RAMOS, and KSHV-positive BC3 and BCBL1 cells, and the mRNA was quantified using Cyation5 nanodrop machine. (**B and C**) BJAB and BCBL1 cells were treated with bromouridine (BrU) and induced with either hypoxia or normoxia for 18 h. The cells were stained with Fluorescein isothiocyanate(FITC) -conjugated primary antibody against BrdU, and the BrU incorporation was measured by flow cytometry. The data were analyzed by FlowJo, and the percentage of FITC-positive cells was plotted. (**D**) BJAB, BL41, RAMOS, BCBL1, BL41-KSHV, BC3, and BJAB-KSHV cells were induced with either hypoxia or normoxia for 24 h, total protein was isolated, and western blot was performed to access the expression of RNA Pol II. Induction of hypoxia was confirmed by the expression of HIF1α, and the band intensity was normalized based on the levels of β-actin. All the experiments were performed twice independently, and the best representative data sets are presented. Results are presented as mean ± s.d. from the three experiments. ***P-value < 0.001; **P-value < 0.01; *P-value < 0.05; and ns, no significance.

Hypoxia is a metabolically unfavorable condition that hinders many cellular processes. To check the effect of hypoxia on the levels of total mRNA in KSHV-positive and negative cells, 10 million cells from two KSHV-positive pleural effusion lymphoma cell lines (BCBL1 and BC3) and two KSHV-negative cell lines (BJAB and Ramos) were either exposed to hypoxia or normoxia for 24 h, and total RNAs were isolated and measured. During hypoxia, there was a significant drop of >50% total in the quantity of RNA in KSHV-negative BJAB and Ramos cells when compared to normoxia. However, in KSHV-positive cells, the levels of RNA showed negligible difference (Fig. 1A). To further confirm the effect of hypoxia on the quantity of total mRNA, BJAB and BCBL1 cells were transfected with a plasmid coding for DsRed, and the cells were induced with either hypoxia or normoxia for 24 h. Post-incubation, the cells were evaluated for the expression of DsRed protein using flow cytometry. We posit that the rate of transcription has a direct effect on the rate of DsRed protein synthesis and that the presence of KSHV will impact the expression of DsRed protein. The results showed a significant increase in the mean fluorescence intensity during hypoxia in KSHV-positive BCBL1 cells when compared to the KSHV-negative BJAB cells (Fig. S1).

To directly elucidate the role of KSHV on transcription efficiency, we used bromouridine (BrU) incorporation assay. BJAB and BJAB-KSHV cells were treated with 25 µm of BrU and induced with hypoxia or normoxia for 18 h. No cellular toxicity was observed at this concentration. Post 18 h, the cells were fixed and treated with anti-BrdU antibody that also recognizes the BrU nucleotide analog (30, 31). The cells were counterstained with FITC-conjugated secondary antibody (Fig. 1B). We hypothesized that the effect of hypoxia on transcription will be reflected in the amount of incorporated BrU. After 18 h of culture in normoxia, both BJAB and BCBL1 cells showed a considerable amount of BrU incorporation in the mRNAs as evident from the almost identical increase in the percentage of FITC-positive cells (Fig. 1C). However, during hypoxia there was a significant difference between BJAB and BJAB-KSHV cells in the number of FITC-positive cells. Nearly 49% of BJAB-KSHV cells were positive compared to only 2% of BJAB cells (Fig. 1C). These experiments clearly point out the differential transcriptional efficiency between BJAB and KSHV-positive BJAB-KSHV cells during hypoxia.

### KSHV protects cellular RNA Pol II from hypoxia-mediated degradation

Hypoxia limits the cellular availability of oxygen forcing the cell toward anaerobic respiration. Anaerobic respiration is energetically unfavorable and slows down essential cellular processes (32). Our initial findings hinted toward a slower rate of transcription during hypoxia. In this section, we determine the effect of hypoxia on the levels of the key transcription factor, RNA Pol II. KSHV-positive cells (BCBL1, BC3, BJAB-KSHV, and BL41-KSHV) and KSHV-negative cells (BJAB, BL41, and RAMOS) were exposed to either hypoxia or normoxia for 24 h, and the total protein lysates were harvested. The levels of RNA Pol II were evaluated by western blot. In KSHV-positive cells, the change in RNA Pol II level during hypoxia is not as significant as that of KSHV-negative cells (Fig. 1D). From these results, we can infer that hypoxia affects the cellular transcription machinery by modulating levels of RNA Pol II and that the presence of KSHV inhibits the effect of hypoxia by rescuing the RNA Pol II levels to maintain transcription in the hypoxic microenvironment.

### KSHV-encoded LANA attenuates hypoxia-induced degradation of RNA Pol II

**Fig 2:**
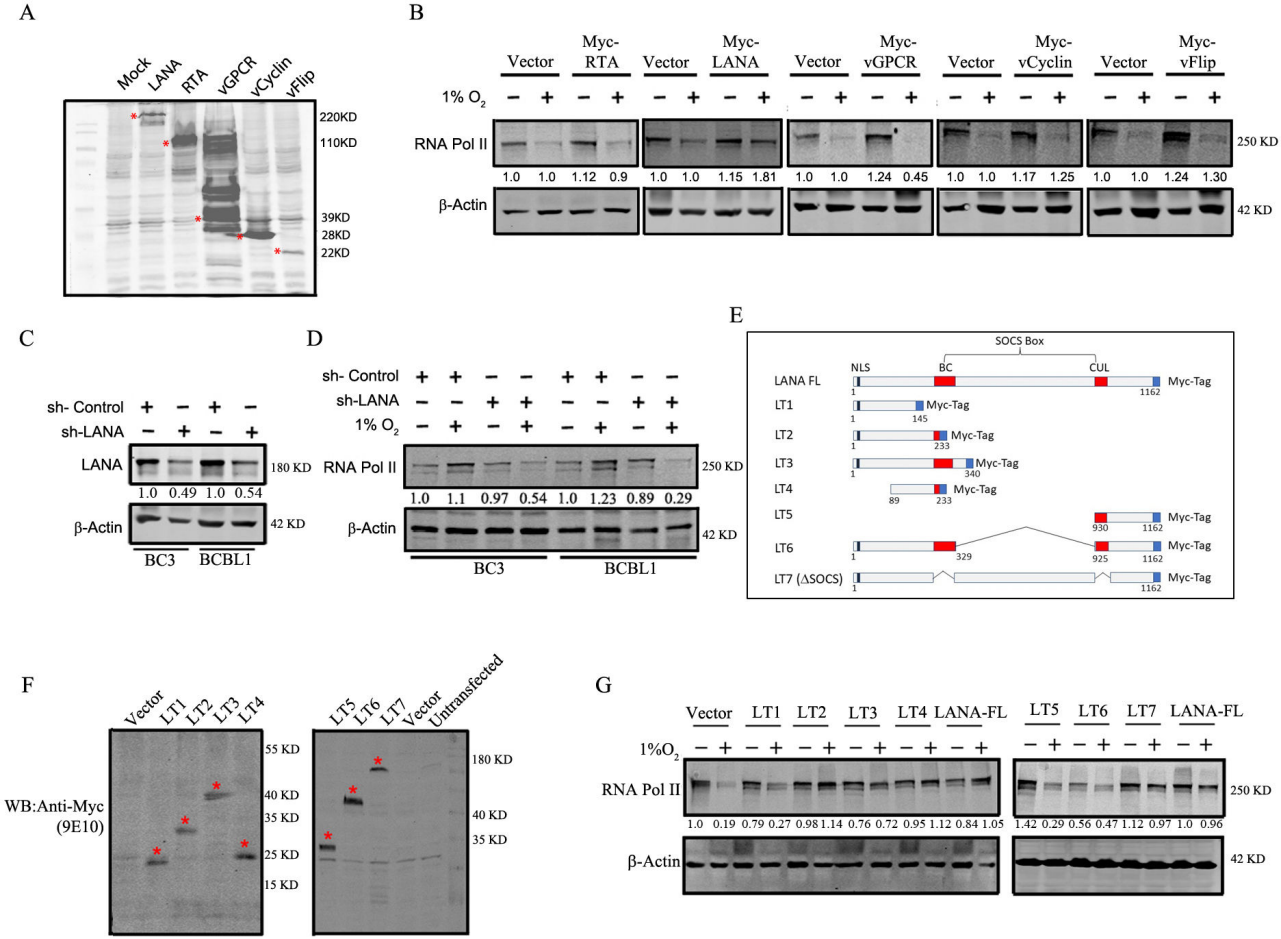
KSHV-encoded LANA inhibits the degradation of RNA Pol II during hypoxia. (**A**) Expression of KSHV-encoded antigens. Mock, Myc-tagged vCyclin, Myc-tagged vGPCR, Myc-tagged LANA, Myc-tagged RTA, or Myc-tagged vFLIP plasmids were transfected into HEK293 cells followed by western blot analysis using anti-Myc antibody. (**B**) KSHV antigen-expressing cells were induced with hypoxia for 24 h followed by western blot to check the expression of RNA Pol II. The blots were normalized based on β-actin. (**C**) Validation of knockdown of LANA in BC3 and BCBL1 cells. (**D**) Effect of knockdown (KD) of LANA in BC3 and BCBL1 cells on RNA Pol II expression during normoxia and hypoxia. BC3 and BCBL1 cells with or without knockdown of LANA were exposed to normoxia or hypoxia for 24 h, and the expression of RNA Pol II was measured by western blot. (**E**) Schematic of the different truncations of Myc-conjugated LANA. (**F**) Conformation of the expression of different truncations of Myc-tagged LANA by western blot. (**G**) Effect of the different truncations of LANA on RNA Pol II during hypoxia and normoxia.

To identify the KSHV-encoded gene responsible for rescuing the levels of RNA Pol II from hypoxia-mediated degradation, we transfected HEK293 cells with expression constructs of KSHV-encoded genes, namely, RTA, latency-associated nuclear antigen (LANA), vCyclin, vFlip, and vGPCR under the control of a heterologous promoter. These genes are known to be differentially expressed under hypoxic conditions. The results showed that the Myc-tagged fusion proteins were all detected by the anti-Myc antibody (Fig. 2A). We then wanted to determine the effects of the expression of these genes in the hypoxic microenvironment. When compared to all the other KSHV-encoded genes expressed, LANA expression demonstrated the most dramatic change in the rescue of the RNA Pol II levels (Fig. 2B). Briefly, a reduction of two- to threefold in the expression of RNA Pol II was observed in cells expressing mock or the other KSHV-encoded genes including RTA, vGPCR, vCyclin, and vFLIP grown under hypoxia. However, this reduction was rescued in cells expressing LANA grown under hypoxia (Fig. 2B).

To validate the involvement of LANA in the process, KSHV-positive BC3 and BCBL1 cells, which expressed LANA, were stably transduced with lentivirus (sh-LANA) to suppress LANA expression (Fig. 2C). These cells were then exposed to either hypoxia or normoxia in culture. The results showed that cells with LANA knockdown (KD) were unable to rescue the levels of the cellular RNA Pol II in hypoxia (Fig. 2D). These findings emphasize a crucial role that LANA plays in rescuing the levels of RNA Pol II in hypoxia potentially through inhibition of its proteasomal degradation.

### Amino terminal domain of LANA is sufficient to confer protection of RNA Pol II in hypoxia

The essential latent gene, LANA, is a multifunctional protein encoded by KSHV. LANA plays a vital role in maintaining the latency and persistence of the viral episome (33). To identify the specific structural motif of LANA responsible for RNA Pol II protection, we generated seven LANA truncations (LT1–LT7) fused with myc epitope in a pA3M expression vector, as shown in Fig. 2E. Following transient exogeneous expression of these constructs in HEK293 cells, the cells were analyzed for the expression of fusion proteins (Fig. 2F). These transiently transfected cells were exposed to hypoxia or normoxia for 24 h in culture, and the expression levels of RNA Pol II were assessed by western blotting. Except for LT1 and LT5, RNA Pol II levels were maintained during hypoxia in all other clones (Fig. 2G). In LT1 and LT5, RNA Pol II was degraded during hypoxic induction. LT2, which corresponds to 1–233 amino acids of the N-terminal, successfully protected RNA Pol II from degradation. Based on these observations, we postulate that 1–233 residues of the N-terminal domain of LANA are critical for this rescue activity.

### Role of LANA and HIF1α in regulation of RNA Pol II levels

**Fig 3:**
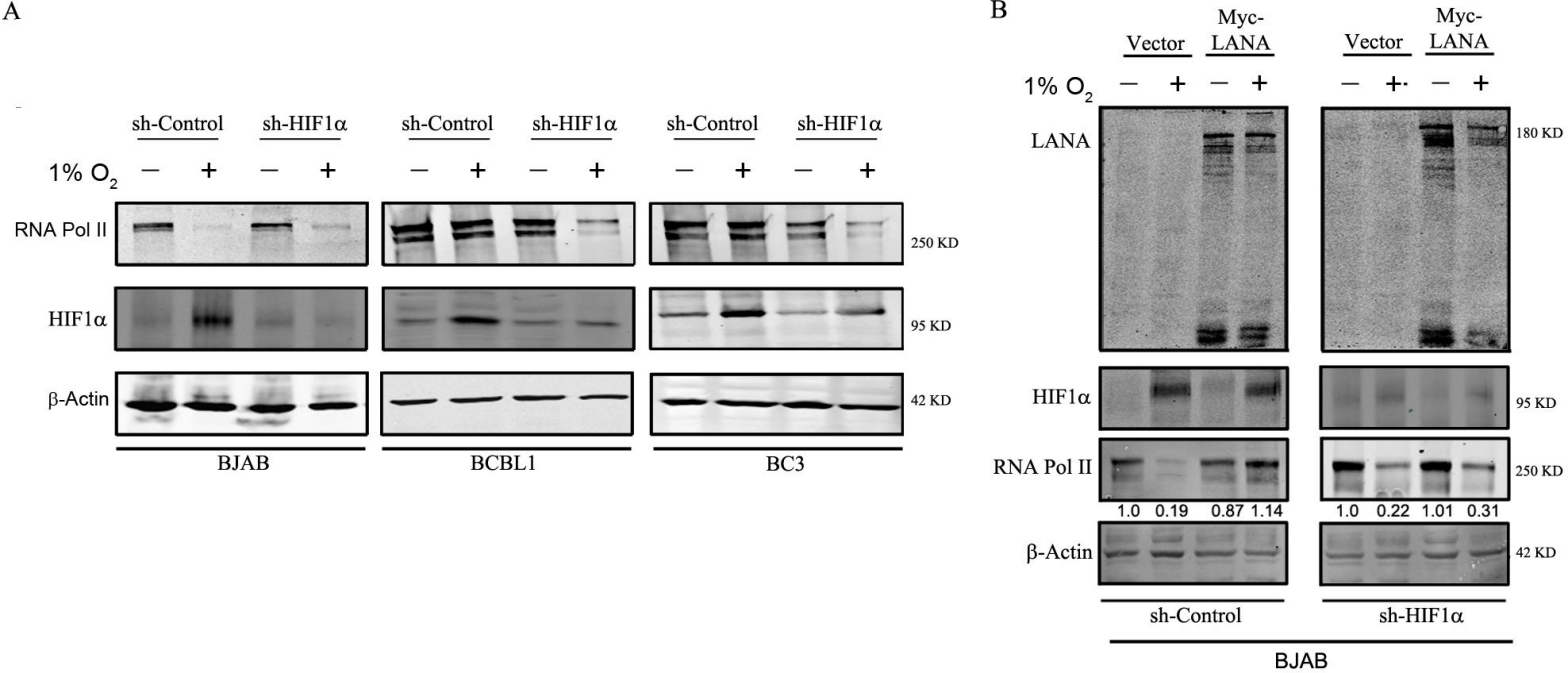
Effect of HIF1α on RNA polymerase during hypoxia. (**A**) BJAB, BC3, and BCBL1 cells were stably knocked down for HIF1α and were exposed to either hypoxia or normoxia for 24 h. The expression of RNA Pol II and HIF1α was assessed by western blot and were normalized based on the expression of β-actin. (**B**) BJAB cells that were either stably transfected with sh-HIF1α or with sh-control were transiently transfected with plasmids expressing Myc-tagged LANA. The expression of LANA, HIF1α, and RNA Pol II was detected by western blot. All these experiments were performed in duplicates, and the best representative blots are presented.

Previous studies from our lab reported that 1–233 amino acid residues of the N-terminal domain of LANA can form a RING finger structure using disulfide linkage that interacts with HIF1α and regulates its localization and function (34). In the above assay, we showed that the N-terminal domain of LANA was sufficient to rescue RNA Pol II from degradation. Therefore, we predicted the probable involvement of HIF1α in this process. To shed light on this, we also knocked down HIF1α in KSHV-negative BJAB cells and KSHV-positive BC3 and BCBL1 cells and incubated them in hypoxia. To ensure proper knockdown and induction of hypoxia, the expression of HIF1α was analyzed by western blot (Fig. 3A). We observed that silencing of HIF1α in BC3 and BCBL1 cells led to lower levels of RNA pol II during hypoxia. There was an approximately twofold reduction of RNA Pol II levels in sh-HIF1α KD cells during hypoxia as compared to sh-control when compared to lane 2 and lane 4 (Fig. 3A). We performed a cell viability assay to further confirm that knockdown of HIF1α did not have a significant effect on the cell viability (Fig. S2A). This provides evidence that HIF1α also plays a crucial role in the differential regulation of RNA Pol II levels. To further determine the requirement of LANA and HIF1α in regulating RNA Pol II levels, BJAB cells were KD for HIF1α and transfected with expression plasmids for myc-LANA and were exposed to hypoxia. The expression of LANA and HIF1α was confirmed by western blot (Fig. 3B). We observed that cells expressing both LANA and HIF1α could rescue RNA Pol II during hypoxia, whereas cells that only expressed LANA were not sufficient for this activity. These findings demonstrated that both LANA and HIF1α are critical for regulating the degradation of RNA Pol II during hypoxia.

### Hypoxia induces the degradation of RNA Pol II through the proteasomal pathway

**Fig 4:**
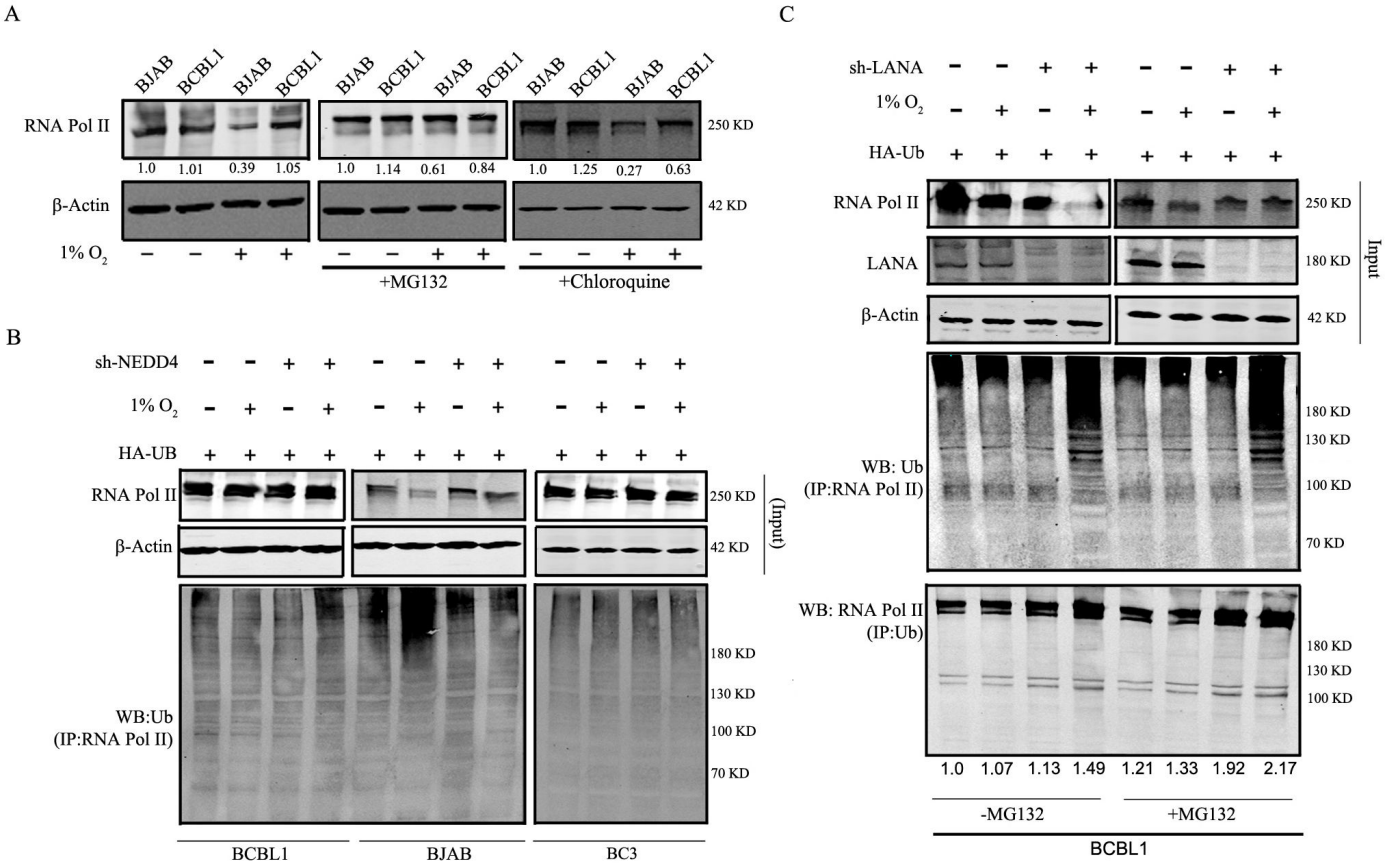
Proteasomal degradation of RNA Pol II during hypoxia. (A) BJAB and BCBL1 cells were either treated with or without MG132 and chloroquine and exposed to hypoxia or normoxia. To determine the levels of RNA Pol II, western blotting was employed. (**B**) BJAB, BC3, and BCBL1 cells were KD for NEDD4 and induced with hypoxia or normoxia. Expression of RNA Pol II was determined by western blot. The whole cell lysate was used to immunoprecipitate RNA Pol II, and western blot (WB) was performed for Ub to check the levels of ubiquitylation of RNA Pol II. (**C**) LANA was KD from BCBL1 cells and was treated with or without MG132 and induced with or without hypoxia. Input levels of RNA Pol II and LANA were estimated by western blot. The lysate was used separately to pull down either RNA Pol II or Ub and blotted for Ub or RNA Pol II, respectively. All these experiments were performed in duplicates, and the best blot is presented.

Previous reports suggest that physiological or chemical stress leading to DNA damage induces polyubiquitylation and subsequent proteasomal degradation of RNA Pol II (35, 36). We showed above that levels of RNA Pol II were dramatically reduced during hypoxia. To elucidate the pathway utilized for RNA Pol II degradation, BJAB and BCBL1 cells were treated with either MG132 or with chloroquine for 24 h and exposed to hypoxia. MG132 and chloroquine are potent inhibitors of proteasomal pathway and lysosomal pathway, respectively. We observed that in BJAB cells, the reduction of RNA Pol II was minimized in the presence of MG132. However, there was no significant change observed in chloroquine treatment group as compared to the untreated control (Fig. 4A). This result demonstrates that under hypoxic conditions, the effect on the RNA Pol II levels is due to its degradation through the proteasomal pathway as treatment with MG132 was able to reverse the loss seen in control cells (Fig. 4A).

Previous reports showed that NEDD4 functions as a E3-ubiquitin ligase and that RNA Pol II is one of its targets, which is polyubiquitinylated and degraded through the proteasomal pathway (37). We investigated the contribution of NEDD4 to RNA Pol II degradation during hypoxia. BJAB, BC3, and BCBL1 cells were knocked down for NEDD4 or transfected with sh-control plasmids. These cells were exposed to hypoxia or normoxia for 24 h, harvested, and the ubiquitination assay was performed. We focused on studying the ubiquitination of RNA Pol II. We performed an immunoprecipitation (IP) for RNA Pol II and analyzed its ubiquitination pattern by western blot analysis. The results clearly showed that in BJAB cells during hypoxia, there was significantly more ubiquitination as compared to normoxia (Fig. 4B). However, KD of NEDD4 predominantly inhibited the ubiquitination of RNA Pol II as seen by a reduction in the untreated sh-NEDD4 lane and minimal change in the treated lane (Fig. 4B). In KSHV-positive cell lines (BC3 and BCBL1), there is no significant alteration in the ubiquitination pattern between sh-NEDD4 and sh-control cells during hypoxia and normoxia (Fig. 4B, left and right panels). These data further support our hypothesis that during hypoxia in KSHV-negative cells, RNA Pol II is polyubiquitinylated by NEDD4 and that leads to its degradation. Whereas in KSHV-positive cells, there is minimal effect seen on RNA Pol II in the presence of NEDD4 during hypoxia due to ubiquitinoylation and is clearly rescued in the KSHV-negative BJAB cells KD for NEDD4 (Fig. 4B).

### Effect of KSHV-encoded LANA on the ubiquitylation of RNA Pol II during hypoxia

We have shown that LANA is essential for the rescue of RNA Pol II from hypoxia-mediated degradation (Fig. 2B). To investigate the role of LANA in the ubiquitination of RNA Pol II, BCBL1 cells were transduced with control lentivirus or with sh-LANA lentivirus to knockdown LANA. The cells were exposed to hypoxia for 24 h. Immunoprecipitation was done using specific antibody against RNA Pol II, and WB was performed for ubiquitin (Fig. 4C). KD of LANA led to a significant decrease in the levels of RNA Pol II and a subsequent increase in the polyubiquitination as evident from the laddering pattern of the WB (Fig. 4C). Notably, MG132 treatment rescued RNA Pol II from proteasomal degradation (Fig. 4C). To further validate this finding, Ub was immunoprecipitated using Ub-specific antibody, and RNA Pol II signals were detected by western blot analysis. We clearly noticed the increase in Ub co-immunoprecipitated RNA Pol II levels in LANA KD cells that were not treated with MG132 (Fig. 4C, bottom panel). There is significantly more Ub-bound RNA Pol II in cells that are not expressing LANA. Furthermore, cells that were treated with MG132 also showed a similar trend, but the overall amount of RNA Pol II coprecipitated was much higher for all the samples as MG132 actively hinders the proteasomal degradation pathway (Fig. 4C, lanes 5–8). We performed a similar experiment with BJAB cells and observed significant degradation of RNA Pol II and subsequently more ubiquitination during hypoxia as compared to normoxia (Fig. S2B). In the presence of MG132, RNA Pol II levels were rescued and there was significantly less laddering (lanes 3 and 4). The data provide clear evidence that RNA Pol II undergoes ubiquitination and subsequent degradation in the absence of LANA and that the E3 ubiquitin ligase NEDD4 is responsible for its ubiquitination.

### Role of LANA, HIF1α, and NEDD4 in the rescue of RNA Pol II from degradation

**Fig 5:**
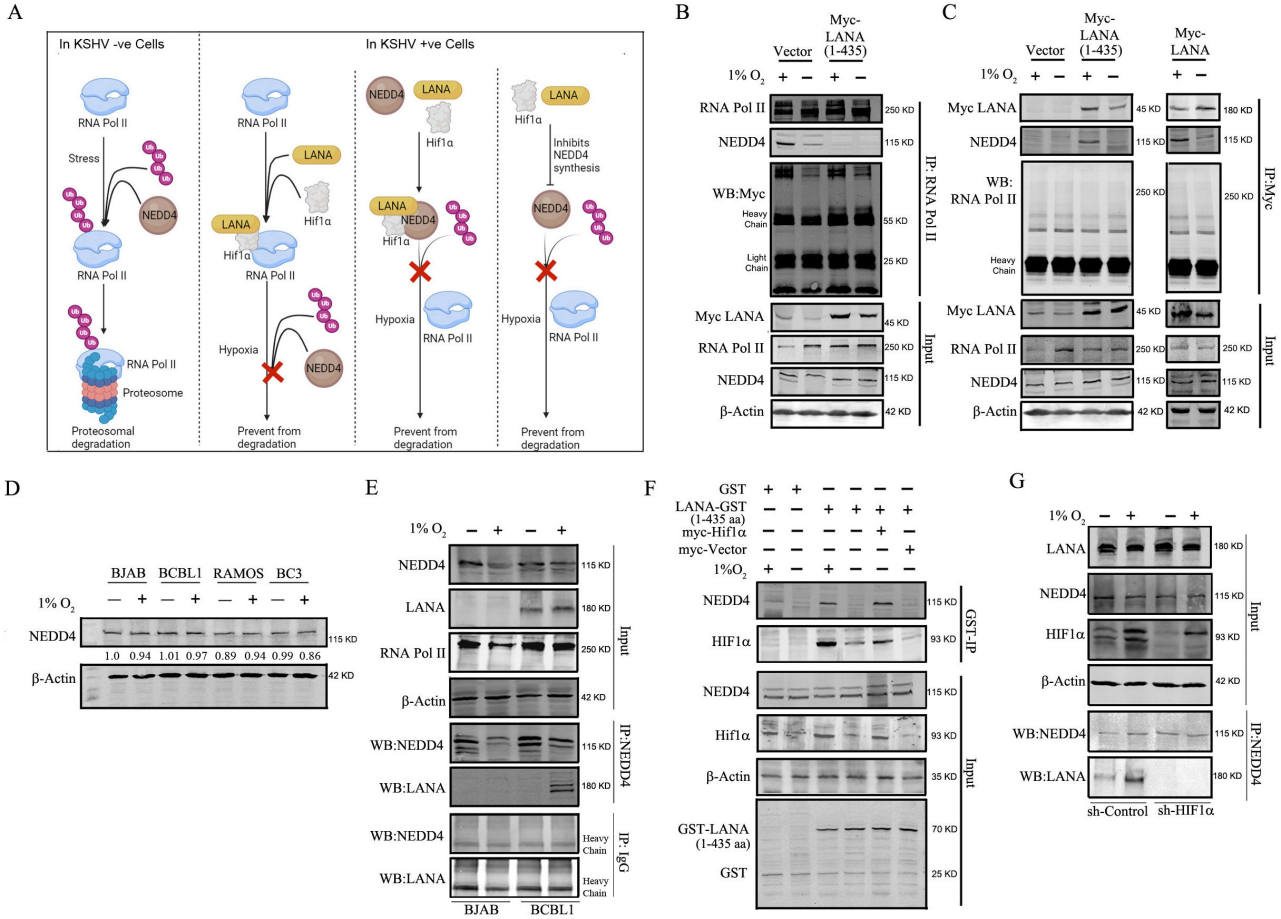
Role of LANA, HIF1α, and NEDD4 in the protection of RNA Pol II from hypoxia-mediated degradation. (**A**) Schematic diagram displaying the probable pathways of protection of RNA Pol II from degradation due to hypoxia. (**B and C**) BJAB cells either transfected with empty vector or Myc-LANA (1–435 aa) or full-length LANA were induced with either hypoxia or normoxia, and the expression of Myc, RNA Pol II, and NEDD4 was measured by western blot. The cell lysates were immunoprecipitated for either RNA Pol II or Myc and blotted for RNA Pol II and NEDD4. (**D**) BJAB, RAMOS, BC3, and BCBL1 cells were induced with or without hypoxia, and NEDD4 expression was monitored by western blot. (**E**) BJAB and BCBL1 cells were induced with or without hypoxia and immunoprecipitated for NEDD4 and checked for LANA and NEDD4 as the interacting partner by western blot. (**F**) GST-tagged truncated LANA (1–435 aa) was expressed in bacteria and purified using glutathione-Sepharose beads. BJAB cells or BJAB cells expressing either Myc-HIF1α or Myc-empty vector were exposed to hypoxia or normoxia, and the total cell lysate was used to perform an *in vitro* pull-down assay. The pulldown was confirmed by western blot. (**G**) ShRNA knockdown of HIF1α in BCBL1 was followed by induction in hypoxia or normoxia, and total protein was isolated for immunoprecipitation of NEDD4. The presence of LANA in the precipitated samples was determined by western blot. All experiments were performed in duplicates, and the best representative blots were presented.

We hypothesize that during physiological stress, RNA Pol II is stalled on the DNA template due to either a shortage of available ATP or the DNA damage induced by the reactive oxygen species generated during hypoxia. The stalled RNA Pol II will be ubiquitinated by NEDD4, which leads to its proteasomal degradation (28). From the previous data, we propose three probable models that can potentially rescue or protect RNA Pol II from hypoxia-mediated degradation (Fig. 5A). First, LANA and HIF1α can directly interact with RNA Pol II blocking the ubiquitylation site and thus conferring protection. Second, LANA and HIF1α interact with NEDD4 and block its polyubiquitylating activity, which spares RNA Pol II from degradation. Lastly, LANA and HIF1α downregulate NEDD4 expression, leading to a decrease in the efficiency of RNA Pol II polyubiquitylation (Fig. 5A).

To address whether LANA interacts with RNA Pol II, we expressed the N-terminal domain of LANA (amino acid residues 1–435) fused to the Myc epitope tag in BJAB cells. Immunoprecipitation was done using anti-RNA Pol II-specific antibody, and binding was evaluated for the presence of Myc-LANA in the IP samples by western blot for the Myc epitope using anti-Myc-specific antibody. We could not detect the presence of Myc-LANA in any of the samples (Fig. 5B). To further validate this observation, we expressed either the N-terminal domain of LANA (amino acid residues 1–435) or the full-length LANA fused to the Myc epitope tag in BJAB cells. The anti-Myc antibody was used for immunoprecipitation, followed by western blot to confirm the presence of RNA Pol II. We observed similar results in that, and we could not detect RNA Pol II in the IP samples (Fig. 5C). These findings strongly support the hypothesis that the amino terminal 1–435 residues of LANA or the full-length LANA and RNA Pol II are not interacting during hypoxia or normoxia.

To investigate whether the presence of KSHV has any effect on the overall expression of NEDD4, western blot for NEDD4 was performed. KSHV-positive BC3 and BCBL1 cells and KSHV-negative BJAB and RAMOS cells were grown in hypoxia or normoxia. No significant difference was observed in the expression levels of NEDD4 in KSHV-positive and KSHV-negative cell lines (Fig. 5D). This experiment clarifies that the presence of KSHV or expression of KSHV-encoded LANA had no effect on the levels of NEDD4.

Next, we investigated whether LANA and HIF1α can interact with NEDD4. To address this, BJAB and BCBL1 cells were induced with hypoxia, NEDD4 was immunoprecipitated, and western blot was performed to detect the presence of LANA. We found that during hypoxia in KSHV-positive cells, LANA co-immunoprecipitated with NEDD4 but not in normoxia (Fig. 5E). We further verified that there was no cross-reactivity by immunoprecipitation with non-specific IgG antibody and could not detect LANA or NEDD4 in the pull-down samples as seen by the western blots (Fig. 5E). This observation led to the conclusion that during hypoxia NEDD4 preferentially forms a complex with LANA. To further confirm this finding, an *in vitro* binding assay was performed in which the GST-tagged N-terminal domain of LANA (1–435 amino acids) was overexpressed in a bacterial system and isolated using glutathione beads. The expression was confirmed by western blot analysis (Fig. 5F). BJAB cells were exposed to hypoxia or normoxia, then lysed, and the whole cell lysates were incubated with GST-LANA (1–435) isolated from bacteria. Immunoprecipitation was performed using an anti-GST antibody and blotted for the presence of NEDD4 and HIF1α. We noted that in the cell lysates of cells grown in hypoxia, NEDD4 and HIF1α were co-immunoprecipitated with GST amino-terminal LANA (Fig. 5F). We also observed a similar result in cells that were in normoxia but overexpressing HIF1α. This experiment clearly points out that both HIF1α and NEDD4 can form a complex with LANA and that is predominantly seen in hypoxia.

We further validated this finding by knocking down HIF1α in BCBL1 cells that were induced with hypoxia. Immunoprecipitation with NEDD4 was performed, and the samples were checked for the presence of LANA. In control cells, we detected LANA as a co-immunoprecipitated partner, while in the HIF1α KD cells, LANA was not detected (Fig. 5G). These experiments clearly demonstrate that LANA forms a tripartite complex with NEDD4 and HIF1α and that this interaction results in the rescue of RNA Pol II from polyubiquitination by NEDD4.

## DISCUSSION

Replication, transcription, and translation processes are critical for the survival of cells, but they rely on a constant supply of ATP. During aerobic respiration, molecular oxygen acts as the final electron acceptor in the electron transport chain, which leads to the production of ATP (38). However, during hypoxia, oxygen deprivation forces cells to shift to anaerobic respiration, resulting in the production of less ATP than aerobic respiration (39). To adapt to this stressed condition caused by hypoxia, cells decrease their catabolic rate (40). As replication, transcription, and translation are all catabolic processes, cells must balance these essential processes to maintain homeostasis during hypoxic conditions.

Our study aimed to explore the differential regulation of transcription in cells infected with KSHV under hypoxic conditions, in comparison to KSHV-negative cells. Hypoxia can initiate the reactivation of a subset of KSHV-positive cells that were previously latent (41), requiring the synthesis of specific virus-encoded structural and non-structural proteins through active transcriptional and translational processes. Our initial data suggest that hypoxia significantly impairs the process of active transcription, but the presence of KSHV somehow rescues this process, as evidenced by the higher total RNA quantity and halogenated ribonucleotide incorporation in KSHV-positive cells. We hypothesized that the lower transcription rate in KSHV-negative cells during hypoxia may be due to the reduced levels of RNA Pol II. Indeed, we observed significantly lower levels of RNA Pol II in KSHV-negative cells compared to KSHV-positive cells during hypoxic stress. We then investigated the role of KSHV antigens in rescuing RNA Pol II from hypoxia and found that LANA plays a crucial role. To confirm the role of LANA, we knocked down LANA in KSHV-positive BC3 and BCBL1 cells and observed that LANA-depleted cells behaved similarly to KSHV-negative cells and were unable to maintain RNA Pol II levels during hypoxia (Fig. 2D). In a previous study, we identified the N-terminal domain of LANA and its interaction with HIF1α as important during hypoxia (34). Here, we further identified that the specific region between 1 and 233 amino acids of the N-terminal domain of LANA is essential for the protection of RNA Pol II from hypoxia-induced proteasomal degradation.

HIF-1 is a transcriptional activator that requires oxygen to function at physiological levels and is regulated by the Von Hippel Lindau E3 ligase. It plays important roles in tumor angiogenesis and mammalian development. HIF1α is composed of a constitutively expressed HIF1β subunit and one of the three subunits, namely HIF1α, HIF2α, or HIF3α (42). The functionality and stability of HIF1α are based on different post-transcriptional modifications, and it is reported to interact with different cellular and viral proteins. Under normoxia, the HIF-1α subunit is rapidly degraded through the ubiquitin-proteasomal degradation pathway (42). However, during hypoxia, different coactivators interact with HIF1α to increase its stabilization. Moreover, different KSHV antigens have previously been reported to regulate or interact with HIF1α (34, 43–45). To investigate the role of HIF1α in protecting RNA Pol II during hypoxia, short hairpin RNA (shRNA) was used to knockdown HIF1α in KSHV-positive BC3 and BCBL1 cells. The results showed that loss of HIF1α resulted in reduced levels of RNA Pol II during hypoxia, indicating the importance of HIF1α in rescuing RNA Pol II (Fig. 3A). To further support our findings, we conducted experiments on cell lines expressing LANA and knocked down HIF1α. Our results showed that the presence of either LANA or HIF1α alone was not sufficient to rescue RNA Pol II.

Hypoxia generates reactive oxygen species (46) and can cause DNA damage (47, 48). During transcription, RNA Pol II stalls at DNA lesions (49), leading to premature termination due to the degradation of RNA Pol II via the ubiquitin-mediated proteasomal pathway (28, 50). We used chloroquine (lysosomal inhibitor) and MG132 (proteasomal inhibitor) to determine the mechanism behind the hypoxic-mediated degradation. We found that only MG132 was unable to rescue RNA Pol II from degradation in KSHV-positive BCBL1 cells. Previously, it has been reported that RNA polymerase II ubiquitylation and degradation are important DNA damage responses, conserved from yeast to humans (51), and identified NEDD4 as the prime E3 ubiquitin ligase that polyubiquitinylates RNA Pol II, leading to its degradation (52). To determine the role of NEDD4, we knocked down NEDD4 and noticed that RNA Pol II degradation was reversed even in the absence of KSHV during hypoxia. We also observed less ubiquitination and subsequently less laddering in the ubiquitination assay in the cells KD for NEDD4. These data were further validated in KSHV-positive cells that were KD for LANA. Furthermore, KD of LANA in BCBL1 cells led to the degradation of RNA Pol II and displayed laddering during hypoxia. These results clearly showed that LANA was essential for the rescue of RNA Pol II from ubiquitination during hypoxia.

This study suggests that LANA, NEDD4, and HIF1α interact to prevent RNA Pol II degradation during hypoxia. Three possible models were proposed, and our findings support the third model. Other potential interactions between LANA and RNA Pol II, as well as the regulation of NEDD4 by KSHV, were investigated but did not show significant evidence to support any of the other two models. However, NEDD4 was found to pull down LANA specifically during hypoxia, indicating complex formation. *In vitro* binding assays confirmed these results. HIF1α was also found to be crucial for the interaction between LANA and NEDD4. Overall, the LANA-NEDD4-HIF1α interaction is essential for protecting RNA Pol II during hypoxia and maintaining its levels for active transcription.

This is an exciting finding as it sheds light on another mechanism by which KSHV can overcome the metabolic stress of hypoxia and reactivate its lytic cycle. The discovery that LANA is involved in protecting RNA Pol II by interacting with HIF1α and NEDD4 is a significant contribution to the understanding of KSHV biology as well as transcription regulation in hypoxia. Further studies are needed to fully elucidate the structural and functional details of this tripartite interaction and how it is regulated in the context of different cellular physiological states. This knowledge could potentially lead to the development of new therapies for KSHV-associated diseases.

## MATERIALS AND METHODS

### Cell culture, plasmid constructs, transfection, and RNA isolation

BJAB, BJAB-KSHV, BC3, BCBL1, and Ramos cells were grown in Roswell Park Memorial Institute (RPMI) medium containing 10% bovine growth serum and penicillin/streptomycin at the recommended concentration and maintained at 37°C and 5% CO_2_. For hypoxia, cells were cultured and incubated in special chambers with 1% O_2_ and 5% CO_2_ and supplemented with N_2_ for 18–30 h.

Myc-tagged LANA, RTA, vCyclin, vFLIP, and vGPCR were constructed in the pA3M vector as described earlier (45). Transfection experiments were performed using jetPRIME reagent (Polyplus Transfection Inc., New York, NY, USA) according to the manufacturer’s protocol. RNAs were isolated from cultured cells by using TRIZOL.

The pA3M-LANA truncated constructs carrying the c-Myc-tagged ORF73 amino-terminal domain containing amino acids (aa) 1–145 (LT1), 1–233 (LT2), 1–340 (LT3) and carboxy-terminal domain (aa 930–1,162 LT5) and the pA3M-LANA truncated constructs carrying Myc-tagged ORF73 aa 89–233 (LT4), aa 1–329, and 925–1,162 (LT6) were constructed as discussed previously (34). The LT7 was generated from full-length LANA with the SOCS region deleted.

### Western blotting and immunoprecipitation

Whole cell lysates were prepared by using RIPA buffer (50 mM Tris, pH 7.6, 150 mM NaCl, 2 mM EDTA, 1% Nonidet P-40) supplemented with protease inhibitors as discussed earlier (45). The detailed specification of the antibodies is listed in Table S1. After blotting with specific primary and IR-conjugated secondary antibodies, the membranes were scanned using an Odyssey scanner (LiCor Inc., Lincoln, NE, USA).

For IP, cells were lysed in RIPA buffer and lysates were pre-cleared with protein A/G agarose beads, and the exact protocol was followed as discussed earlier (44). Approximately, 5%–10% of the sample was used as input control depending on target protein.

### Lentiviral-mediated gene silencing

For the lentivirus-mediated knockdown of LANA, HIF1α, and NEDD4, the shRNA sequence (Table S2) was respectively inserted into pGIPZ vector according to the manufacturer’s instructions (Open Biosystem, Inc, Huntsville, AL, USA), A 21-mer oligonucleotide sh-control that had no significant homology to any known human mRNA in the databases was cloned in the same vector and used as control. For the production of lentivirus, pGIPZ clones along with packaging and helper plasmids were transfected into HEK293T cells as described earlier (45).

### Chloroquine and MG132 treatment

The cytotoxicity of chloroquine and MG132 was determined by treating cells with varying concentrations of these chemicals (0, 10, 25, 50, and 100 µM of chloroquine and 0.625, 1.25, 2.5, 5, and 10 µM of MG132). For 24 h, chloroquine was used at a concentration of 50 µM, and 5 µM was used as a maximum concentration of MG132 to treat cells. At 24 h post-transfection, the culture medium was replaced with a fresh medium containing either chloroquine or MG132 for 24 h and exposed to the indicated time of hypoxia or normoxia.

### Flow cytometric analysis

To analyze the transcriptional efficiency, cells were cultured in media containing BrU and exposed to hypoxia or normoxia for 18 h. The cells were washed twice with ice-cold PBS, fixed using 4% paraformaldehyde for 20 min at room temperature, and perforated by 0.1% saponin solution with 1% bovine serum albumin (BSA) for 20 min. Cells were then stained with anti-BrdU primary antibody (1:100) for 4 h in 1% BSA solution at 4°C with rotation at 30 rpm. For detection using flow cytometry, the cells were then incubated with FITC-conjugated (1:200) secondary antibody for 1 h in 1% BSA at room temperature with rotation at 30 rpm. The cells were then acquired using the BD FACS LSR Fortessa and analyzed using a trial version of FLOWJO software.

### GST protein preparation and pull-down assay

*Escherichia coli* BL21 (DE3) cells were transformed with the plasmid constructs for each GST fusion protein. Single colonies were picked and grown, induced with isopropyl β- d-1-thiogalactopyranoside (IPTG), and the GST-fused proteins were isolated as described earlier (53).

## SUPPLEMENTAL MATERIAL

Fig. S1 mbio.02774-23-s0001.tif KSHV modulates hypoxia-induced retardation of metabolic pathways.

Fig. S2 mbio.02774-23-s0002.tif Effect of knockdown of HIF1α on cell viability during normoxia and hypoxia.

Supplemental Materials mbio.02774-23-s0003.pdf Supplemental tables and figure legends.

ASM does not own the copyrights to Supplemental Material that may be linked to, or accessed through, an article. The authors have granted ASM a non-exclusive, world-wide license to publish the Supplemental Material files. Please contact the corresponding author directly for reuse.
