# Supplementary figures and images for: KSHV-encoded LANA bypasses transcriptional block through the stabilization of RNA Pol II in hypoxia
Source: mBio. 2023 Dec 14;15(1):e02774-23. doi: 10.1128/mbio.02774-23 (PMC10790784; doi:10.1128/mbio.02774-23)

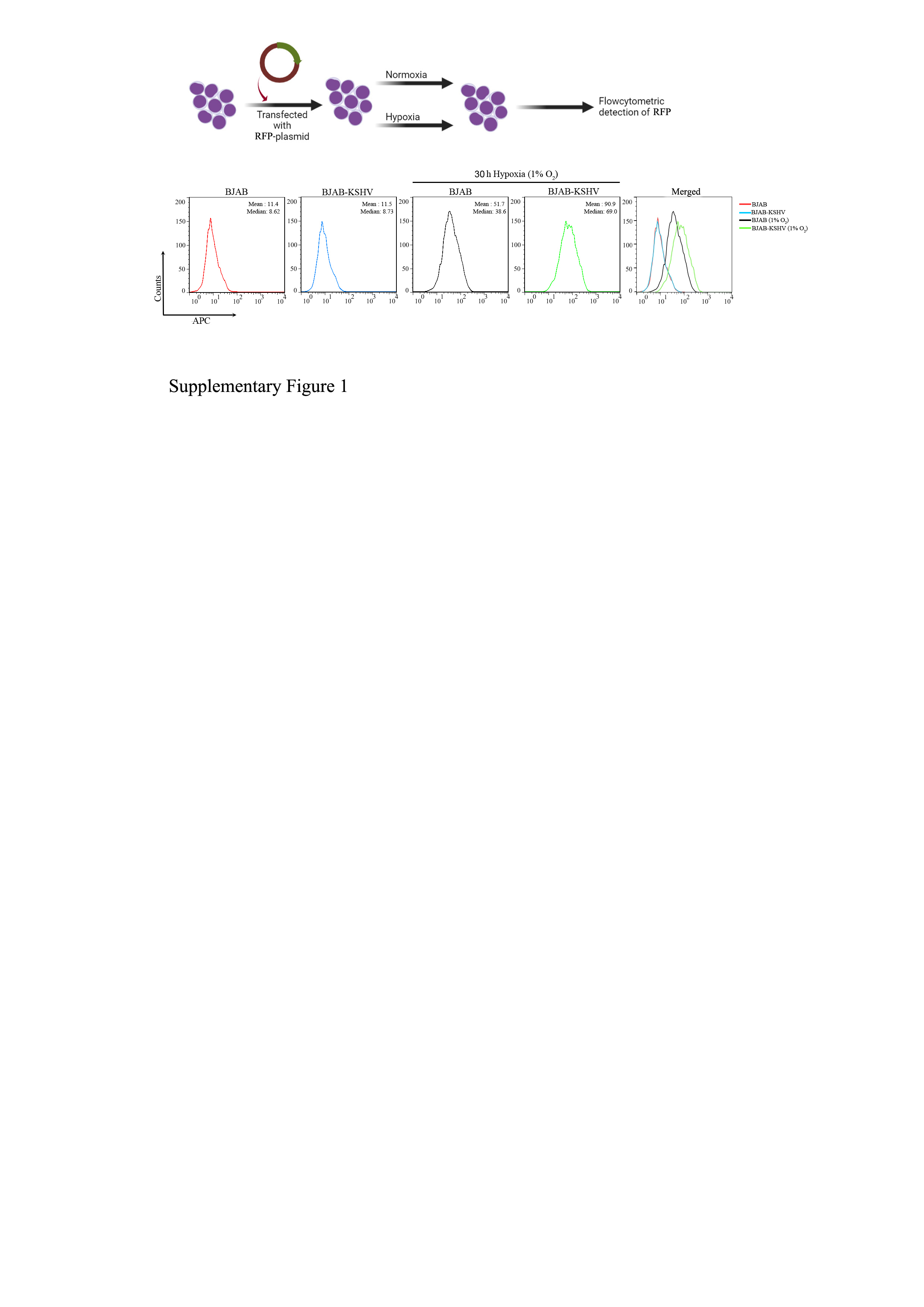

Supplement: Fig. S1 — KSHV modulates hypoxia-induced retardation of metabolic pathways. [file mbio.02774-23-s0001.tif]

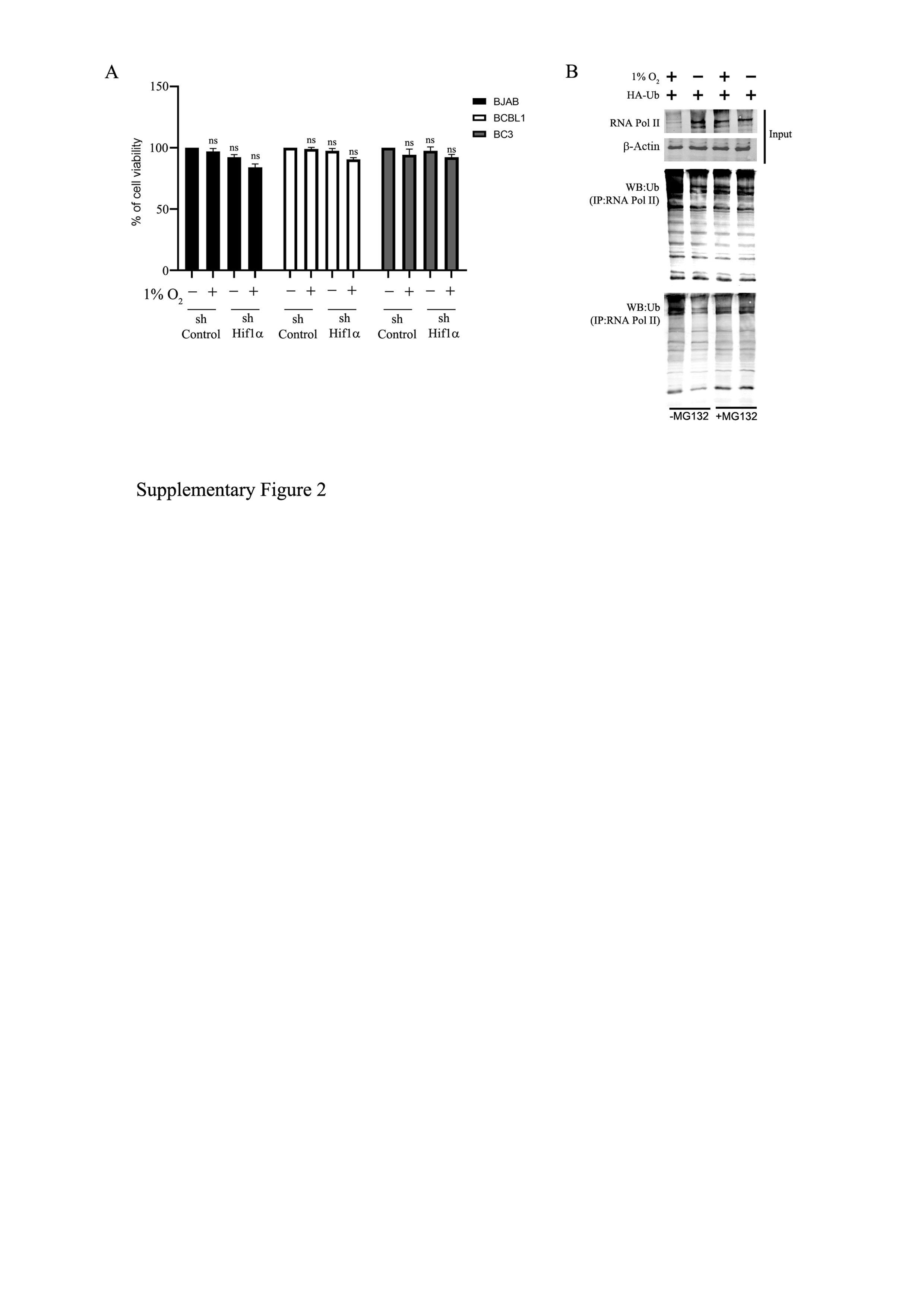

Supplement: Fig. S2 — Effect of knockdown of HIF1α on cell viability during normoxia and hypoxia. [file mbio.02774-23-s0002.tif]
