# Supplementary material for: KSHV-encoded LANA bypasses transcriptional block through the stabilization of RNA Pol II in hypoxia
Source: mBio. 2023 Dec 14;15(1):e02774-23. doi: 10.1128/mbio.02774-23 (PMC10790784; doi:10.1128/mbio.02774-23)
Supplement: Supplemental Materials — Supplemental tables and figure legends. [file mbio.02774-23-s0003.pdf]

Supplementary Information

Supplementary Figure legend

Supplementary Figure 1. KSHV modulates hypoxia induced retardation of metabolic pathways.

BJAB and BCBL1 cells were transfected with DsRed plasmid and induced with hypoxia or normoxia for 24 h. The cells were washed, and flow cytometric analysis was performed for the detection of the expression of DsRed. The data were analyzed by FlowJo and graphically presented.

Supplementary Figure 2.

A. Effect of knock down of HIF1α on cell viability during normoxia and hypoxia. B. Effect of MG132 on RNA Pol II levels in BJAB cells induced with either hypoxia or normoxia. BJAB cells were treated with or without MG132 and ubiquitination assay was performed.

Supplementary Table 1:

Supplementary Table 1: List of antibodies

Antibody Name	Company	Catalog No
Anti BrdU Antibody	Santa Cruz Biotechnology	sc-32323
Anti-RNA polymerase II Antibody, clone CTD4H8	Millipore Sigma	05-623
HIF-1α Antibody	Novus Biologicals	NB100-105
β-Actin	Santa Cruz Biotechnology	sc-47778
Anti-Myc (9E10)	Generated from hybridomas	25222-1-AP
Ubiquitin Antibody (P4G7)	Santa Cruz Biotechnology	sc-53509
NEDD4 Polyclonal antibody	Proteintech	21698-1-AP

Supplementary Table 2: Sequences of primers

shRNA Name	Sequence
NEDD4.1	CGCCTTGACTTACCTCCATAT
NEDD4.2	CCGGAGAATTATGGGTGTCAA
HIF1α .1	CCGCTGGAGACACAATCATAT
HIF1α .2	GTGATGAAAGAATTACCGAAT
